# A pragmatic and safe way of inducing immunotolerance in selected peanut allergic children: a single centre experience

**DOI:** 10.1186/2045-7022-5-S3-P150

**Published:** 2015-03-30

**Authors:** Emily Stenke, Claire Cullinane, Deirdre Daly, Jonathan O B Hourihane

**Affiliations:** 1 Department of Paediatrics, Cork University Hospital, Cork, Ireland

## Background

Peanut oral immunotherapy is both safe and effective in the treatment of children with peanut allergy but study protocols demand large resource commitment, limiting their adoption in routine clinical practice. We present 8 selected peanut allergic children who have achieved either full tolerance or an increased threshold to peanuts following a home based, low dose introduction of peanuts.

## Methods

8 children (age 4-12 years) with suspected peanut allergy failed an oral food challenge at the final dose (n=7) or second-last dose (n=1) of peanut ingestion. They were commenced on daily home ingestion of small amounts of peanut (starting dose 1 peanut or equivalent) with incremental doses. All patients were equipped with adrenaline auto-injectors and trained in their use.

## Results

5 patients had previously reacted to peanut (4 by ingestion, 1 with topical exposure) with first exposure between the ages of 14-24 months. 3 had never been accidentally exposed to peanut. 7 children had eczema, 4 asthma and 5 had other food allergies (milk, egg, hazelnut, lentil). In all 8 patients, peanut allergy was confirmed by OFC. Skin prick wheal size ranged from 4-9mm. All patients had had at least one SPT >= 6mm. SpIgE to peanut and araH2 at any time ranged from <0.10- 9.49 kU/L and <0.1- 6.28 kU/L respectively; just prior to starting the home peanut ingestion levels ranged from <0.1- 5.33 kU/L, SpIgE to araH2 <0.10- 6.28 kU/L. All 8 children failed OFCs to peanut; at the final dose of >= 8grams in 7 patients and at 4 grams in 1 patient. OFC reactions were gastrointestinal (n=2), mouth itch (n=2)), urticarial (n=4), facial flushing (n=2), facial swelling (n=2) and hoarse voice (n=1). No patient needed treatment with adrenaline.

4 children have subsequently had 2 negative OFCs; 1 challenge while on peanut dosing and 1 six weeks after stopping. 4 children await OFCs. Seven children remain on home peanut with no adverse events. One child developed abdominal pain and vomited after accidentally increasing the amount of peanut ingested and has subsequently discontinued the programme for taste reasons.

## Conclusion

In patients with high dose reactivity to peanut during OFC and low IgE based tests, oral immunotherapy may be safely introduced at home.

